# Immunization coverage and predictive factors for complete and age-appropriate vaccination among preschoolers in Athens, Greece: a cross- sectional study

**DOI:** 10.1186/1471-2458-13-908

**Published:** 2013-10-02

**Authors:** Ioanna D Pavlopoulou, Koralia A Michail, Evangelia Samoli, George Tsiftis, Konstantinos Tsoumakas

**Affiliations:** 1 Pediatric Clinic, "P & A Kyriakou" Children’s Hospital, National and Kapodistrian University of Athens, Faculty of Nursing, Athens, Greece; 2 Postgraduate Program, National and Kapodistrian University of Athens, Faculty of Nursing, Athens, Greece; 3 Department of Hygiene, Epidemiology and Medical Statistics, National and Kapodistrian University of Athens Medical School, Athens, Greece; 4 Department of Preventive Medicine, Municipal Nursery of Athens, Athens, Greece

**Keywords:** Vaccination coverage, Age-appropriate immunization, Predictive factors, Conjugated pneumococcal vaccine, Conjugated meningococcal C vaccine, Varicella vaccine, Greece

## Abstract

**Background:**

In Greece, several new childhood vaccines were introduced recently but were reimbursed gradually and at different time points. The aim of this study was to assess immunization coverage and identify factors influencing complete and age-appropriate vaccination among children attending public nurseries in the municipal district of Athens.

**Methods:**

A cross-sectional study, using stratified sampling was performed. Immunization history was obtained from vaccination booklets. Demographic and socioeconomic data were obtained from school registries and telephone interviews. Vaccination rates were estimated by sample weighted proportions while associations between complete and age-appropriate immunization and potential determinants by logistic regression analysis.

**Results:**

A total of 731 children (mean age: 46, median: 48, range: 10–65 months) were included. Overall immunization coverage with traditional vaccines (DTP, polio, Hib, HBV, 1st dose MMR) was satisfactory, exceeding 90%, but the administration of booster doses was delayed (range: 33.7- 97.4%, at 60 months of age). Complete vaccination rates were lower for new vaccines (Men C, PCV7, varicella, hepatitis A), ranging between 61-92%. In addition, a significant delay in timely administration of Men C, PCV7, as well as HBV was noted (22.9%, 16.0% and 27.7% at 12 months of age, respectively). Child’s age was strongly associated with incomplete vaccination with all vaccines (p< 0.001), while as immigrant status was a predictor of incomplete (p=0.034) and delayed vaccination (p<0.001) with traditional vaccines. Increasing household size and higher maternal education were negatively associated with the receipt of all and newly licensed vaccines, respectively (p=0.035).

**Conclusions:**

Our findings highlight the need to monitor uptake of new vaccines and improve age- appropriate administration of booster doses as well as early vaccination against hepatitis B. Immigrant status, increased household size and high maternal education may warrant targeted intervention.

## Background

Immunization is one of the most effective public health tools, leading to reduced infant and childhood morbidity and mortality, caused by vaccine preventable diseases worldwide [1-4]. However, outbreaks of vaccine preventable diseases continue to occur in developed countries, and this may be attributed to unimmunized or under-immunized subpopulations despite high overall vaccination coverage [5-9]. Several underlying factors for suboptimal vaccine uptake have been described, but are complex and present diversities among different countries, and population groups [10-16]. It is, therefore, essential to monitor vaccination coverage regularly and identify reasons that contribute to delay or non- vaccination at local levels, in order to develop appropriate strategies to address the needs of susceptible populations.

In Greece, vaccines included in the National Immunization Programme (NIP) are universally recommended and are provided free of charge to all resident children, including immigrants. Children can be vaccinated in the private sector or primary health centers and health insurance clinics, the latter two at no additional cost. Vaccinations are recorded in health booklets, but there is no routine nationwide monitoring and no official vaccine reminder system.

As in most developed countries, the Hellenic immunization schedule has become more complex in the last decade, with the introduction of new vaccines and recommendation of more doses of existing vaccines. It is noteworthy that reimbursement of new vaccines has occurred recently, mostly in a gradual and non- simultaneous process. Therefore, compliance with newly recommended vaccines and associated factors has not been well documented.

Several local and one nationwide study assessing vaccination coverage with old vaccines (DTP/DTaP, OPV/IPV, MMR, Hib and hepatitis B) in pre- school and first grade grammar school children have been published in our country but have not addressed factors influencing under immunization [17-20]. According to a national survey performed in 2006 among 6- year olds, various socioeconomic factors, rather than parental attitudes and beliefs towards vaccines, were identified as predictors for incomplete and delayed immunizations [21]. Since then, new multivalent vaccines, as well as conjugated meningococcal C (Men C), 7-valent pneumococcal (PCV7), varicella (Var) and hepatitis A vaccines (HAV), licensed between 1999 and 2004, were gradually incorporated in the Hellenic NIP. Men C and PCV7 were partially reimbursed in 2005, Var in 2006, and all others, including HAV, were fully compensated in 2008. Moreover, a significant increase in the immigrant population, including children, originating from countries with limited vaccine resources, has occurred during the last years [22,23]. At present, data concerning immunization uptake of newer vaccines and related determinants remain insufficient.

The aim of this study was to assess immunization coverage and identify factors affecting complete and age-appropriate vaccination among a large population of children attending public nurseries in the municipal district of Athens, after the introduction of a full reimbursement policy of new vaccines.

## Methods

A cross-sectional study was undertaken using stratified sample design in all public nurseries of the Municipality of Athens, Greece during the school year 2010–2011. The above area consists of 98 nursery schools with a total of 5,401 registered pupils of middle and low income families and is further divided into seven subregions. Nurseries were equally stratified into all seven municipal subregions. These were further subdivided by the child’s gender into boys and girls in order to better reflect the children’s gender distribution by region. On the day of population recording, a total of 4,165 pupils were present, but only 3,399 were considered for further evaluation, those whose parents had presented a health booklet. The minimum required sample size was estimated, using Snedecor & Cochran’s formula for random sampling from finite populations [24]. Final sample consisted of 731 children (368 boys and 363 girls) with 90% power and +/- 10% estimate of true proportion.

Children were randomly sampled from each stratum, as opposed to the entire 3,399 children, in order to extract the number of subjects estimated by sample stratification, followed by personal telephone interviews from children’s guardians. In the case of incorrect phone number or non-reply, following a total of five repeated attempts, simple random sampling was repeated, in order to complete the originally calculated number of 731 individuals. The sampling stopped once the minimum sample size was achieved.

All school teachers invited to participate, were informed through interactive sessions organized by the investigators at the beginning of the school year. Following an explanatory letter, all parents/guardians of children of the selected schools were asked to take part in the study by presenting their child’s vaccination booklet and by answering to a subsequent telephone interview.

### Data collection

A structured questionnaire completed by the investigators was used. Basic demographic data were collected from school registries on the day of school visit. Detailed vaccination history and use of combination vaccines were obtained from vaccination booklets. Parental/guardian attitudes towards immunization and additional information were gathered on a second occasion by telephone interview.

### Definitions of vaccination status

Children were considered completely and timely immunized according to the 2006 NIP guidelines (see Additional file 1: Table S1). Definitions of complete and age- appropriate immunization are presented in Table 1. We decided to use the 2006 NIP as reference because the majority of the pupils belonged to this age group. Assessment of immunization coverage against hepatitis A was performed separately, since this vaccine was introduced and fully reimbursed in 2008, for all infants older than 12 months.

**Table 1:** Definitions of complete and age- appropriate vaccination

**Vaccine**	**Complete vaccination**	**Age appropriate vaccination at the age of**		
		**12 months**	**24 months**	**60 months**
DTP/DTaP	3 doses by the age of 12 months, or	3 doses	4 doses	5 doses
	4 doses by the age of 24 months, or			
	5 doses by the age of 60 months			
OPV/IPV	2 or 3 doses by the age of 12 months plus 1 dose ≥ 12 months, or	3 doses	3 or 4 doses	4 or 5 doses
	3 doses by the age of 24 months or			
	4 or 5 doses by the age of 60 months			
Hib	2 or 3 doses by the age of 12 months plus	2 or 3 doses	≥ 3 doses	≥ 3 doses
	1 dose between ≥ 12 – 23 months, or			
	2 doses between 12 – 24 months, or			
	1 dose at the age of ≥ 24 months.			
MMR	1 dose between ≥ 12 < 48 months, or	-	≥ 1 dose	2 doses
	2 doses between ≥ 48 – 60 months			
HBV	≥ 3 doses	3 doses	3 doses	3 doses
Men C	2 doses < 12 months, or	2 or 3 doses	≥ 3 doses	≥ 3 doses
	1 dose ≥ 12 months			
PCV7	3 doses by the age of 12 months, plus	3 doses	4 doses	4 doses
	1 dose ≥ 12 – 23 months, or			
	2 doses from the age of 7 – 12 months, plus			
	1 dose ≥ 12 – 23 months, or			
	2 doses between 12 – 23 months, or			
	1 dose ≥ 24 months			
Var	1 dose between ≥ 12 < 48 months, or	-	≥ 1 dose	2 doses
	2 doses between ≥ 48 – 60 months			
HAV	≥ 2 doses at ≥ 12 months	-	2 doses	2 doses

DTP/DTaP: diphtheria, tetanus, pertussis/ acellular pertussis vaccine; OPV/IPV: live attenuated/inactivated polio vaccine; HBV: hepatitis B vaccine; MMR: measles, mumps, rubella vaccine; Hib: haemophilus influenzae type b vaccine; Men C: conjugated meningococcal C vaccine; PCV7: conjugated pneumococcal 7-valent vaccine; Var: varicella vaccine; HAV: hepatitis A vaccine.

### Ethical considerations

The study protocol was approved by the ethics committee of the University of Athens, Faculty of Nursing and the review board of the Municipal Nursery of Athens. Informed consent was implied when parents provided their child’s health booklet and participated in the telephone interview that followed.

### Statistical analysis

In order to examine associations between complete and age-appropriate vaccination with potential determinants we, initially, applied simple cross-tabulations and the X^2^-test. Weighted proportions were used to account for the sampling design.

Consequently, we applied multiple logistic regression models [25] including the child’s actual age at interview (in years, continuously), father’s nationality (Greek versus other), mother’s education (high school or less versus college/university), number of children in the family (categorically as: 1, 2, 3 or more), use of multivalent vaccines (3- or 4-valent versus 5- or 6-valent). Additional variables included the impact of vaccine cost and the insurance coverage on the decision for vaccination by the parents (both as yes versus no), place of vaccination (private pediatrician versus public health clinic/ insurance doctor) and finally the social security [Social Security Institute (IKA) versus other]. The choice of the variables entered in the models was based on the results from an initial exploratory analysis, possible confounding effects and likelihood ratio backward selection. Special attention was paid in order not to include confounders that were highly correlated (as indicated from the univariate analysis through either X^2^ test or the Spearman correlation coefficient). Possible determinants of interest, such as the impact of vaccines’ cost on the decision for vaccination, were entered in the models irrespectively of statistical criteria. For comparability reasons, in the interpretation of the associations we applied the same model when considering complete or age- appropriate vaccination. When analyzing vaccination status for the three newer vaccines, the influence of vaccine’s cost was not considered as a possible determinant since there was no variability as parents of all age appropriate vaccinated children responded that it did not affect their decision. Statistical analysis was performed by SPSS for Windows, version 16.0 (SPSS, Chicago, IL, USA).

## Results

From October 2010 through November 2011, information concerning vaccination history was obtained from a total of 3,399 health booklets available on the day of school visit (participation rate 81.6%). The final stratified sample consisted of 731 children, and the response rate was 85%. The mean age of the individuals on the day of sampling was 46 months (median, 48 months; range: 10–65 months).

Table 2 illustrates selected demographic characteristics of the study population. Of the 731 pupils, 504 (68.9%) were of Greek nationality, 126 (17.2%) were Albanian, and the remaining 101 (13.8%) originated from other foreign countries. The majority of children (61%) were insured with Social Security Institute (IKA). Interestingly, a relatively high proportion of mothers (41%) were college or university graduates.

**Table 2:** Selected demographic characteristics of the sample

	**N (%)**
*Gender*	
Male	368 (51.7)
Female	363 (48.3)
*Actual age, (months)*	
≤ 24	27 (3.7)
25 - 48	363 (50.3)
49 - 65	341 (45.9)
*Father’s nationality*	
Greek	504 (68.9)
Albanian	126 (17.2)
Other immigrant	101 (13.8)
*Number of children*	
1	183 (14.7)
2	417 (57.4)
≥ 3	131 (17.9)
*Type of insurance*	
IKA	452 (61.1)
Other	279 (38.9)
Maternal education*	
High school or less	433 (59)
College/University	297 (41.1)

IKA: Social Security Institute; * One mother deceased.

Table 3 shows parental attitudes towards vaccines. Nearly two thirds of the parents decided to vaccinate their child in order to offer protection or to prevent disease. Most parents (66.6%) decided to vaccinate their children at insurance or public health clinics. Although most guardians denied that vaccine cost could be a drawback (91.7%), a high proportion (66.4%) admitted that insurance reimbursement would have a positive impact on their decision to vaccinate their child.

**Table 3:** Parental attitudes towards vaccines

	**N (%)**
*Reasons for vaccinating*	
Protection, immunity, good health	354 (47.9)
Prevention of infections	162 (22.6)
Pediatrician’s advice	103 (14.4)
It is compulsory	112 (15.1)
*Would vaccine cost influence your decision to vaccinate your child?*	
Yes	75 (10.3)
Probably yes	21 (2.9)
Maybe	118 (15.9)
Probably not	56 (7.7)
No	461 (63.3)
*Does insurance reimbursement influence your decision?*	
Yes	487 (66.4)
No	244 (33.6)
*Where do you vaccinate you child?*	
Private pediatrician*	239 (33.4)
Insurance doctor or clinic†	485 (65.7)
Public health clinic†	7 (0.9)

* Doctor’s fee only, †No additional costs.

### Immunization coverage

Observed sample sizes and weighted proportions for both complete and age- appropriate immunization coverage are presented in Table 4. Complete immunization rates for diphtheria, tetanus, pertussis, poliomyelitis, Hib and hepatitis B were satisfactory overall, exceeding 94%. However, complete overall coverage for MMR was lower, reaching 63.7%. Likewise, age- appropriate immunization rates for the aforementioned vaccines were satisfactory at the age of 12 months, except for hepatitis B, but gradually decreased thereafter. Thus, hepatitis B vaccine uptake did not exceed 27.7% at 12 months but increased with age reaching 86.1% at 24 and 97.4% at 60 months of age.

**Table 4:** Number and weighted proportions for age- appropriate and complete immunization of the study population

	**Age appropriate immunization at:**			**Complete immunization**
**Vaccine**	**12 months**	**24 months**	**60 months**	**(n=731)**
	**(n=731)**	**(n=704)**	**(n=56)**	
	**N (%)**	**N (%)**	**N (% )**	**N (%)**
DTP/DTaP	696 (95.4)	594 (84.8)	19 (33.7)	688 (94,2)
OPV /IPV	629 (86.8)	602 (86.2)	22 (38.8)	723 (98.9)
Hib	688 (94.2)	559 (80.0)	37 (65.6)	691 (94.5)
MMR	-	660 (90.5)	20 (35.9)	462 (63.7)
HBV	202 (27.7)	605 (86.1)	55 (97.4)	688 (94.3)
MenC	164 (22.9)	111 (16.1)	7 (12.4)	671 (91.9)
PCV7	113 (16.0)	66 (9.8)	2 (4.0)	537 (73.1)
Var	-	430 (60.4)	17 (30.1)	435 (61.1)
HAV	-	6 (0.9)	41 (71.7)	450 (62.0)
All 5 traditional vaccines* (DTP/DTaP, OPV/ IPV, Hib, MMR, HBV)	163 (22.6)	417 (60.1)	4 (6.8)	414 (56.9)
All 3 new* (PCV7, Var, MenC)	62 (8.8)	35 (5.2)	1 (2.0)	327 (44.2)

DTP/DTaP: diphtheria, tetanus, pertussis/ acellular pertussis vaccine; OPV/IPV: live attenuated/inactivated polio vaccine; HBV: hepatitis B vaccine; MMR: measles, mumps, rubella vaccine; Hib: haemophilus influenzae type b vaccine; Men C: conjugated meningococcal C vaccine; PCV7: conjugated pneumococcal 7-valent vaccine; Var: varicella vaccine; HAV: hepatitis A vaccine.

* For age-appropriate vaccination at 12 months, the MMR vaccine is excluded from the traditional vaccines denominator and the HAV is excluded from the new vaccines.

Among children eligible for varicella vaccination, 435 [61.1%; 95% confidence interval (CI): 57.5%- 64.7%] were completely immunized, whereas 671 (91.9%; 95% CI: 89.9%- 93.9%) were fully immunized with Men C, 537 (73.1%; 95% CI: 69.9%-76.3%) with PCV7 and 450 (62%; 95% CI: 60.7%- 67.7%) with HAV. In contrast, age- appropriate immunization with the above vaccines was significantly lower, ranging between 4 and 71%, the lowest rates being observed among those vaccines that were gradually reimbursed (Men C and PCV7) when compared to the ones that were compensated simultaneously to their implementation (Var, HAV).

Finally, a total of 414 (56.9%) children (95% CI: 53.3%- 60.5%) were fully immunized with all five traditional vaccines (DTP/DTaP, polio/IPV, Hib, HBV and MMR), whereas 327 (44.2%) children (95% CI: 40.2%- 47.8%) were completely immunized with all three recently introduced vaccines (PCV7, Men C, Var).

### Multiple logistic regression analysis

Table 5 presents multiple logistic regression- derived odds ratios (ORs) and corresponding 95% CIs for the association between sample characteristics and complete and age- appropriate immunization for all five traditional vaccines (DTP/DTaP, IPV/OPV, Hib, MMR and HBV). Factors negatively associated with complete immunization coverage with all of the above vaccines, were the child’s increasing age (p< 0.001) and foreign ethnicity (p= 0.034). In addition, ethnicity seemed to play a significant role on age- appropriate vaccination since Greek children had higher odds to be vaccinated at the age of 24 months than immigrant children (OR: 2.57; 95% CI: 1.71-3.87, p< 0.001). Moreover, age- appropriate vaccination rates increased significantly with the use of multivalent vaccines both at the ages of 12 (p<0.001) and 24 months (p<0.001). Finally, children whose parents expressed negative attitudes concerning vaccine cost, presented lower rates of timely immunization (statistically significant at the age of 24 months, OR: 0.51, 95% CI: 0.28-0.92, p=0.025).

**Table 5:** Multiple logistic regression derived odds ratios (and 95% Confidence Intervals) for the association between sample characteristics and immunization with all 5 traditional vaccines (DTP/DTaP, OPV/IPV, MMR, Hib, HBV)

	**Complete immunization (n=731)**		**Age appropriate immunization at**			
			**12 months (n=731)**		**24 months (n=704)**	
	**OR (95% CI)**	**p**	**OR (95% CI)**	**p**	**OR (95% CI)**	**p**
*Actual age*	0.37 (0.30-0.45)	<0.001	0.83 (0.68-1.01)	0.058	0.85 (0.69-1.05)	0.850
*Father’s nationality*						
Other	Reference					
Greek	1.56 (1.04-2.36)	0.034	0.98 (0.61-1.59)	0.930	2.57 (1.71-3.87)	<0.001
*Number of children*						
1	Reference					
2	1.02 (0.69-1.50)	0.932	1.64 (1.03-2.62)	0.038	0.95 (0.63-1.42)	0.796
3+	0.79 (0.48-1.32)	0.378	1.49 (0.83-2.69)	0.181	0.63 (0.37-1.06)	0.627
*Maternal education*						
≤12 years	Reference					
>13 years	0.84 (0.59-1.18)	0.308	0.99 (0.68-1.46)	0.977	0.88 (0.61-1.27)	0.880
*Use of multivalent vaccines*						
≤ 4-valent	Reference					
5- or 6-valent	1.30 (0.92-1.83)	0.138	3.00 (1.91-4.72)	<0.001	3.19 (2.26-4.51)	<0.001
*Impact of vaccine cost on the decision to vaccinate*						
No	Reference					
Yes	0.88 (0.49-1.58)	0.657	0.85 (0.43-1.70)	0.651	0.51 (0.28-0.92)	0.025
*Impact of insurance reimbursement on the decision to vaccinate*						
No	Reference					
Yes	0.94 (0.64-1.37)	0.741	1.06 (0.70-1.60)	0.800	0.99 (0.67-1.45)	0.949
*Place of vaccination*						
Privately	Reference					
Insurance clinic / Public health clinic	1.17 (0.78-1.76)	0.440	0.97 (0.61-1.55)	0.907	0.88 (0.58-1.33)	0.543
*Type of insurance*						
IKA	Reference					
Other	0.97 (0.63-1.48)	0.876	1.39 (0.87-2.22)	0.165	1.26 (0.82-1.94)	0.296

DTP/DTaP: diphtheria, tetanus, pertussis/ acellular pertussis vaccine; OPV/IPV: live attenuated/inactivated polio vaccine; HBV: hepatitis B vaccine; MMR: measles, mumps, rubella vaccine; Hib: haemophilus influenzae type b vaccine.

IKA: Social Security Institute.

Multiple logistic regression results assessing complete and age-appropriate immunization with all three newly introduced vaccines (Men C, PCV7, Var) are presented in Table 6. As shown, increasing age remained a negative predictor for complete (OR: 0.77; 95% CI: 0.65-0.91, p=0.002) and timely immunization at 12 (OR: 0.47; 95% CI: 0.35-0.64, p<0.001) and 24 (OR: 0.45; 95% CI: 0.29-0.71, p<0.001) months of age. Use of multivalent vaccines had a positive impact on age-appropriate immunization at 12 months (OR: 3.46; 95% CI: 1.51-7.92, p=0.003) while as increased household size (≥ 2 children) was negatively associated with on time administration of vaccines (OR: 0.41; 95% CI: 0.19-0.90, p=0.026) at 24 months. Finally, children of mothers with higher academic education presented lower odds of age-appropriate immunization with all three newly introduced vaccines (OR: 0.42; 95% CI: 0.19-0.94, p=0.035) at 24 months. Results from univariate logistic regression models corresponding to the associations in Tables 5 and 6 are presented in as additional data [see Additional file 2: Tables S2 and S3] for comparability reasons.

**Table 6:** Multiple logistic regression derived odds ratios (and 95% Confidence Intervals) for the association between sample characteristics and immunization with all 3 newer vaccines (MenC, PCV7, Var)

	**Complete immunization (n=731)**		**Age appropriate immunization at**			
			**12 months (n=731)**		**24 months (n=704)**	
	**OR (95% CI)**	**p**	**OR (95% CI)**	**p**	**OR (95% CI)**	**p**
*Actual age*	0.77 (0.65-0.91)	0.002	0.47 (0.35-0.64)	<0.001	0.45 (0.29-0.71)	0.001
*Father’s nationality*						
Other	Reference					
Greek	1.29 (0.88-1.90)	0.188	2.10(0.91-4.86)	0.082	2.95 (0.91-9.50)	0.071
*Number of children*						
1	Reference					
2	0.91 (0.64-1.31)	0.621	0.58 (0.31-1.09)	0.092	0.41 (0.19-0.90)	0.026
3+	0.77 (0.48-1.22)	0.265	0.42 (0.18-1.01)	0.053	0.27 (0.08-0.89)	0.031
*Maternal education*						
≤12 years	Reference					
>13 years	0.73 (0.53-1.02)	0.061	0.68 (0.38-1.22)	0.198	0.42 (0.19-0.94)	0.035
*Use of multivalent vaccines*						
≤ 4-valent	Reference					
5- or 6-valent	1.09 (0.78-1.50)	0.630	3.46 (1.51-7.92)	0.003	2.24 (0.88-5.68)	0.090
*Impact of insurance reimbursement on the decision to vaccinate*						
No	Reference					
Yes	0.95 (0.67-1.34)	0.764	1.22 (0.65-2.28)	0.538	1.28 (0.57-2.90)	0.551
*Place of vaccination*						
Privately	Reference					
Insurance clinic / Public health clinic	1.39 (0.95-2.03)	0.086	0.64 (0.32-1.30)	0.216	0.56 (0.23-1.36)	0.199
*Type of insurance*						
IKA	Reference					
Other	1.13 (0.77-1.66)	0.545	1.67 (0.84-3.34)	0.144	1.69 (0.70-4.11)	0.244

Men C: conjugated meningococcal C vaccine; PCV7: conjugated pneumococcal 7-valent vaccine; Var: varicella vaccine.

IKA: Social Security Institute.

## Discussion

The present study provides important information regarding immunization coverage with traditional and new vaccines, as well as predictive factors for complete and age-appropriate vaccination of preschool children visiting public nurseries of the municipal district of Athens. Our results show that overall immunization rates and vaccination with primary series of traditional vaccines were satisfactory, except for hepatitis B, but the administration of booster doses was delayed. However, overall vaccination coverage was lower for newer vaccines, the most critical finding being the significant delay in administering the primary series of Men C and PCV7. The use of multivalent vaccines was a positive contributor in timely administration of all vaccines while a negative effect was noted with increasing household size. Child’s increasing age was strongly associated with incomplete vaccination with all vaccines, while as immigrant status was a predictor of incomplete and delayed administration of all traditional vaccines, only. Finally, higher maternal education was associated with delayed administration of all newly licensed vaccines.

Regular assessment of vaccination coverage in a given country is a significant tool to improve the effectiveness of vaccination programs, but monitoring of vaccine uptake at a local level is also required in order to prevent vaccine preventable disease outbreaks due to susceptible subgroups [7,26-28]. Moreover, apart from monitoring coverage, it is necessary to identify characteristics or factors, associated with under- vaccination in order to develop appropriate strategies to increase compliance with immunization schedules [10,11,29].

According to our findings, rates of complete overall vaccination coverage of preschoolers against diphtheria, tetanus, pertussis, poliomyelitis, *Haemophilus influenzae* type b, as well as hepatitis B, were proven satisfactory, exceeding 94%. As expected, overall uptake of Hib and HBV was proven higher than recorded before [19,21], possibly due to the widespread use and reimbursement of multivalent vaccines, along with the continuous training of primary care providers about the importance of early protection against hepatitis B. Estimated vaccination coverage with the newer vaccines Men C, PCV7, varicella, and HAV was lower, varying between 61 and 92%, and this may be explained by the recent introduction of these vaccines in the Hellenic NIP and their even more recent full reimbursement policy, only since 2008.

Age- appropriate immunization for older vaccines, except for HBV, was satisfactory; however, this was only true for primary series, with rates inversely associated as age advanced, indicating a delay in administration of booster doses. Efforts should be made to increase timely vaccination with the first dose of MMR, which did not exceed 90.5% at 24 months, and, even more so, to promote earlier administration of the second dose, as only 63% of our individuals had received their booster at the age of 5 years. The latter can be supported by the continuing periodic measles outbreaks in our country due to under immunized indigenous and immigrant population [7,30]. A remarkable result of this study, was the low rate of complete vaccination coverage against hepatitis B before the age of 12 months (27.7%), which has been a consistent finding in other studies in our country [17,19,20]. A possible explanation could be the lack of repayment of the hexavalent vaccine by the nation’s largest social security fund (ΙΚΑ), considering also that the majority of our population was insured with this fund.

In terms of the newest Men C, PCV7 and varicella vaccines, timely vaccination was also found to diminish with age. Nevertheless, the most striking finding was the unacceptably small percentage of children immunized by schedule against *S. pneumoniae* and *N. meningitidis* serogroup C, before the age of 12 months. This lack of compliance could be attributed to the high market price of the above vaccines and the unfavorable refund policy at the time of their introduction, pushing parents and caregivers to delay vaccination in order to reduce the number of required doses and therefore, cost. In contrast, on time vaccination against varicella and hepatitis A was higher, possibly as a result of the simultaneous NIP introduction with reimbursement.

Although achieving a high overall childhood vaccination coverage is important, on time administration of vaccine doses deserves special attention as delays may pose a significant risk for infectious diseases to young infants and children [31,32]. It has been emphasized that delayed administration of the first dose may jeopardize timely administration of the following vaccines [33]. Proposed solutions to address this problem include the creation of reminder systems through the mail or telephone, in addition to adequate training of, health workers and parents, even during pregnancy and the neonatal period [26,33,34]. In view of the recently introduced nationwide electronic drug prescription policy by all health professionals in our country, development of immunization registries appears feasible. Moreover, informing new parents in maternity hospitals about the importance of vaccination before discharge, could contribute significantly to timely administration of the first vaccine dose.

We found that the child’s age remained a strong predictor for incomplete immunization with older and newer vaccines. Foreign ethnicity was significantly associated both with incomplete and delayed administration of all five traditional vaccines. Immigrant status as well as belonging to other minority groups (Roma, various religious groups) have been described as factors leading to suboptimal vaccination worldwide and, therefore, the need for targeted intervention in the aforementioned groups has been underlined [21,35,36]. Interestingly, in the present study, foreign born children presented lower vaccination rates only for traditional vaccines, when compared to native individuals, possibly indicating immunization trends in their countries of origin. In contrast, vaccination coverage with newer vaccines did not differ, and this could be indicative of improved compliance in a controlled environment of their receiving country. In light of the increasing inflow of refugee population from countries of poor socioeconomic status in Greece, the need for ongoing surveillance and targeted intervention is evident.

As described by other investigators [21,37,38], increasing family size was associated with suboptimal, while as use of multivalent formulations with increased compliance for all vaccines, in our population. In addition, higher maternal education was a negative contributor to timely immunization with new vaccines. This was in accordance with a number of reports describing that parents of higher educational level may present lower compliance to vaccine recommendations as a result of their access to various information sources, increasing their concern about vaccine safety [39-41].

Finally, we were not able to draw any firm conclusions on the negative impact of cost on the decision of parents to vaccinate their children. Nevertheless, the increasing proportions of vaccination coverage among our study population in comparison to previously described rates [19] indicates that insurance reimbursement had a positive impact, a conclusion also validated by our model findings, although not to a statistically significant degree. The insignificant impact of cost on immunization in our study may be partly explained by the small percentage of guardians responding positively to the question of whether cost would be a deciding factor for them (8.3%), but also by the retrospective collection of information, comparing current parental beliefs towards vaccines that had already been administered. However, the importance of state’s funding policy has been clearly associated with vaccination coverage in a number of studies, as out-of- pocket costs have been inversely correlated with optimal vaccine administration [42-45]. In view of the economic crisis in our country, an increasing number of children have no insurance for recommended vaccines and visit, therefore, public health centers to receive immunization. In order to prevent immunization gaps, it is essential to improve vaccine delivery in public facilities, including schools, as well as maintaining full reimbursement of all necessary vaccines.

### Study limitations and strengths

The present study had a number of limitations. It was conducted locally, among families of low and middle income and is, therefore, not representative of the whole country. Moreover, the municipal nurseries of Athens could be considered as a supervised environment since the immunization records of all students are tested upon registration and yearly thereafter. Although this could overestimate vaccination coverage, it must be emphasized that non- compliance to complete immunization gaps is not a criterion to deny school entry. Nevertheless, the above article provides significant evidence regarding immunization, collected accurately through health booklets, in the nation’s largest city hosting the majority of native and immigrant population. Therefore, it could be used as a model for designing strategies in order to improve quality and effectiveness of vaccination programs on a local basis.

## Conclusions

Our results highlight the need to monitor uptake of newly introduced vaccines and to develop an effective vaccine reminder system, in order to encourage appropriate administration of booster doses, as well as timely administration of hepatitis B vaccine. Considering the recent introduction of a nationwide electronic drug prescription policy, the implementation of immunization registries in order to improve the quality of vaccination programs, appears feasible. Immigration, increasing number of siblings and high maternal education were factors associated with suboptimal immunization and may warrant targeted intervention. The support of public health facilities, including schools, as well as the maintenance of a full reimbursement policy, are of paramount importance as gaps in insurance benefits, in view of the economic crisis, could result in missed vaccination opportunities in our country.

## Competing interests

The authors declare that they have no competing interest.

## Authors’ contributions

**IDP:** study conception and design, drafting of manuscript, critical revisions for important intellectual content, supervision, **KAM:** study design, data collection, drafting of manuscript, **ES:** statistical expertise, data analysis, critical revisions **GT:** data collection, technical support, **KT:** supervision, administrative support. All authors read and approved the final manuscript.

## Pre-publication history

The pre-publication history for this paper can be accessed here:

http://www.biomedcentral.com/1471-2458/13/908/prepub

## Supplementary Material

Additional file 1: Table S1 2006 National immunization programme recommendations. Click here for file

Additional file 2: Table S2 Univariate logistic regression derived odds ratios (and 95% Confidence Intervals) for the association between sample characteristics and immunization with all 5 traditional vaccines (DTP/DTaP, OPV/IPV, MMR, Hib, HBV). **Table S3**. Univariate logistic regression derived odds ratios (and 95% Confidence Intervals) for the association between sample characteristics and immunization with all 3 newer vaccines (MenC, PCV7, Var). Click here for file
