# Supplementary material for: Immunization coverage and predictive factors for complete and age-appropriate vaccination among preschoolers in Athens, Greece: a cross- sectional study
Source: BMC Public Health. 2013 Oct 2;13:908. doi: 10.1186/1471-2458-13-908 (PMC3850659; doi:10.1186/1471-2458-13-908)
Supplement: Additional file 1: Table S1 — 2006 National immunization programme recommendations. [file 1471-2458-13-908-S1.pdf]

Table S1 2006 National immunization programme recommendations

Vaccine	Age of vaccination and doses
DTP/DTaP	5 doses at 2, 4, 6, 15–18 months, 4–6 years
IPV/OPV	4 doses at 2, 4, 6–18 months, 4–6 years
Hib	4 doses at 2, 4, 6, 12–15 months
MMR	2 doses at 12–15 months, 4–6 years
HBV	3 doses at 2, 4, 6–18 months or at birth, 1–2, 6–18 months
MenC	3 doses at 2, 4, 15–18 months
PCV7	4 doses at 2, 4, 6, 12–18 months
Var	2 doses at 12–18 months, 4–6 years
HAV‡	2 doses at 12 months to 18 years

DTP/DTaP: diphtheria, tetanus, pertussis/ acellular pertussis vaccine; OPV/IPV: live attenuated/inactivated polio vaccine; HBV: hepatitis B vaccine; MMR: measles, mumps, rubella vaccine; Hib: haemophilus influenzae type b vaccine; Men C: conjugated meningococcal C vaccine; PCV7: conjugated pneumococcal 7-valent vaccine; Var: varicella vaccine; HAV: hepatitis A vaccine; ‡ Introduced and fully reimbursed in 2008.
