# Supplementary material for: Immunization coverage and predictive factors for complete and age-appropriate vaccination among preschoolers in Athens, Greece: a cross- sectional study
Source: BMC Public Health. 2013 Oct 2;13:908. doi: 10.1186/1471-2458-13-908 (PMC3850659; doi:10.1186/1471-2458-13-908)
Supplement: Additional file 2: Table S2 — Univariate logistic regression derived odds ratios (and 95% Confidence Intervals) for the association between sample characteristics and immunization with all 5 traditional vaccines (DTP/DTaP, OPV/IPV, MMR, Hib, HBV). Table S3. Univariate logistic regression derived odds ratios (and 95% Confidence Intervals) for the association between sample characteristics and immunization with all 3 newer vaccines (MenC, PCV7, Var). [file 1471-2458-13-908-S2.pdf]

Table S2 Univariate logistic regression derived odds ratios (and 95% Confidence Intervals) for the association between sample characteristics and immunization with all 5 traditional vaccines (DTP/DTaP, OPV/IPV, MMR, Hib, HBV)

	Complete immunization (n=731)	Age appropriate immunization at the age of	
		12 months (n=731)	24 months (n=704)
Actual age	0.37 (0.30-0.45)[b]	0.77 (0.64-0.92)[a]	0.79 (0.65-0.95)[a]
Father's nationality			
Other	Reference		
Greek	1.51 (1.10-2.08)[a]	1.38 (0.93-2.05)	3.33 (2.39-4.64)[b]
Number of children			
1	Reference		
2	1.01 (0.71-1.44)	1.57 (1.00-2.45)[a]	1.01 (0.70-1.45)
≥ 3	1.04 (0.66-1.64)	1.52 (0.87-2.66)	0.71 (0.44-1.13)
Maternal education			
High school or less	Reference		
College/University	0.95 (0.70-1.27)	1.24 (0.87-1.77)	1.36 (1.00-1.86)[a]
Use of multivalent vaccines			
≤ 4-valent	Reference		
5- or 6-valent	1.86 (1.37-2.52)[b]	3.36 (2.17-5.18)[b]	3.74 (2.72-5.16)[b]
Impact of vaccine cost on the decision to vaccinate			
No	Reference		
Yes	0.95 (0.57-1.60)	0.81 (0.42-1.55)	0.40 (0.23-0.68)[b]
Impact of insurance reimbursement on the decision to vaccinate			
No	Reference		
Yes	1.01 (0.73-1.37)	1.13 (0.78-1.64)	0.76 (0.55-1.05)
Place of vaccination			
Private pediatrician	Reference		
Insurance doctor or clinic/ Public health clinic	1.15 (0.84-1.56)	1.21 (0.83-1.77)	0.86 (0.62-1.18)
Type of insurance			
IKA	Reference		
Other	1.27 (0.94-1.72)	1.68 (1.18-2.38)[a]	2.10 (1.52-2.90)[b]

DTP/DTaP: diphtheria, tetanus, pertussis/ acellular pertussis vaccine; OPV/IPV: live attenuated/inactivated polio vaccine; HBV: hepatitis B vaccine; MMR: measles, mumps, rubella vaccine; Hib: haemophilus influenzae type b vaccine.

[a] p< 0.05, [b] p< 0.001; IKA: Social Security Institute.

Table S3 Univariate logistic regression derived odds ratios (and 95% Confidence Intervals) for the association between sample characteristics and immunization with all 3 newer vaccines (MenC, PCV7, Var)

	Complete immunization (n=731)	Age appropriate immunization at	
		12 months (n=731)	24 months (n=704)
Actual age	0.75 (0.64-0.89)[a]	0.46 (0.35-0.60) [b]	0.42 (0.28-0.65)[b]
Father's nationality			
Other	Reference		
Greek	1.25 (0.91-1.72)	2.80 (1.35-5.78)[a]	3.57 (1.24-10.25)[a]
Number of children			
1	Reference		
2	0.98 (0.69-1.39)	0.59 (0.33-1.05)	0.44 (0.21-0.91)[a]
≥ 3	0.93 (0.59-1.46)	0.54 (0.24-1.21)	0.36 (0.12-1.11)
Maternal education			
High school or less	Reference		
College/University	0.85 (0.63-1.15)	0.98 (0.58-1.67)	0.58 (0.27-1.22)
Use of multivalent vaccines			
≤ 4-valent	Reference		
5- or 6-valent	0.24 (0.92-1.68)	5.03 (2.26-11.21)[b]	3.05 (1.25-7.46)[a]
Impact of insurance reimbursement on the decision to vaccinate			
No	Reference		
Yes	1.06 (0.77-1.44)	1.06 (0.61-1.84)	0.97 (0.47-1.98)
Place of vaccination			
Private clinic	Reference		
Insurance doctor or clinic / Public health clinic	1.35 (0.99-1.85)	0.94 (0.54-1.64)	0.74 (0.67-1.48)
Type of insurance			
IKA	Reference		
Other	1.30 (0.96-1.75)	1.82 (1.08-3.07)[a]	1.58 (0.80-3.13)

Men C: conjugated meningococcal C vaccine; PCV7: conjugated pneumococcal 7-valent vaccine; Var: varicella vaccine.
[a] p< 0.05, [b] p< 0.001; IKA: Social Security Institute.
